# Response Characteristics of Biological Soil Crusts Under Different Afforestation Measures in Alpine Sandy Land

**DOI:** 10.3390/biology14050532

**Published:** 2025-05-11

**Authors:** Shaobo Du, Huichun Xie, Gaosen Zhang, Feng Qiao, Guigong Geng, Chongyi E

**Affiliations:** 1 College of Geographical Sciences, Qinghai Normal University, Xining 810008, China; wo827288809@163.com; 2 Qinghai Forest Ecosystem Observation and Research Station in the Southern Qilian Mountains, Haidong 810500, China; 3 Key Lab of Medicinal Animal and Plant Resources of Qinghai-Tibetan Plateau in Qinghai Province, Qinghai Normal University, Xining 810008, China; qiaofnm@163.com; 4 College of Life Sciences, Qinghai Normal University, Xining 810008, China; 5 Key Laboratory of Extreme Environmental Microbial Resources and Engineering, Northwest Institute of Eco-Environment and Resources, Chinese Academy of Sciences, Lanzhou 730000, China; gaosenzhang@hotmail.com; 6 Qinghai Academy of Agriculture and Forestry Sciences, Qinghai University, Xining 810016, China; genggg-298@163.com

**Keywords:** biological soil crust, alpine sandy land, response characteristics, bacterial community structure, sand control measures

## Abstract

Desertification, which may lead to land degradation, is a significant global ecological issue. Biological soil crusts (BSCs) can play a role in sand fixation, carbon sequestration, and the improvement in soil functions in the ecological restoration of sandy soil. Therefore, elucidating the responses of BSCs to afforestation measures in alpine sandy areas is necessary to guide vegetation configuration in sandy ecosystems and enhance the effectiveness of sand fixation measures to prevent desertification. Herein, we determined the physicochemical properties and enzyme activities of bare sand (no crust) and algal and moss crusts collected from four sites subjected to different afforestation measures, including *Salix cheilophila* + *Populus simonii* (WLYY), *Salix psammophila* + *S. cheilophila* (SLWL), *Artemisia ordosica* + *Caragana korshinskii* (SHNT), and *C. korshinskii* (NT80) plantations. High-throughput sequencing was also employed to analyze bacterial community structure in BSCs. The results revealed that fine particle contents in algal and moss crusts were higher than in bare sand. During the succession from bare sand to algae to moss crust, their enzymatic activities and water and nutrient contents tended to increase. And the diversity of bacterial communities changed little in the SLWL sample points, while the richness showed a trend of first decreasing and then increasing, but bacterial community richness and diversity first decreased and then increased at the other sites. Among the four measures, SLWL enhanced nutrient contents, enzyme activities, and bacterial community richness and diversity in BSCs relatively more effectively. Alkaline-hydrolyzable nitrogen and soil organic matter were the key factors impacting bacterial community structures in BSCs under the four afforestation measures. From the perspective of BSCs, the results can provide a reference for the prevention and control strategies of other alpine sandy soils.

## Simple Summary

Herein, we examined the responses of two types of biological soil crusts under different afforestation measures in alpine sandy land. The results revealed that the enzyme activities and fine particle and nutrient contents in both crust types were higher than in bare sand under four different afforestation measures. As the soil transformed from bare sand to algal crust and then to moss crust, the abundance and diversity of their bacterial communities initially decreased and then increased. Among the afforestation measures, *Salix psammophila* + *Salix cheilophila* plantation led to higher levels of enzyme activities, fine particle and nutrient contents, and bacterial community richness and diversity in both crust types compared to other measures. Alkaline-hydrolyzable nitrogen and soil organic matter contents were the most critical physicochemical factors affecting bacterial community structures in the crusts in the study area. Sucrase and alkaline phosphatase activities also correlated significantly with the relative abundances of the dominant bacterial phyla. *S. psammophila* + *S. cheilophila* plantation is more suitable for crust development.

## 1. Introduction

Desertification is a process of declining land productivity in arid, semi-arid, and some humid areas caused by the combined effects of drought, wind erosion, surface sandification, and human interference. It has now been listed as one of the world’s major environmental problems [1,2]. China is among the countries affected by severe land desertification, particularly in its northern regions, where land desertification is extensive and expanding rapidly, thus attracting widespread concern [3]. During the process of desertification, the water-holding capacity of the surface continuously weakens, and soil fertility constantly erodes, which restricts the growth of vegetation. This vicious cycle further intensifies the trend of land desertification, causing significant negative impacts on regional ecological balance and economic development [4,5]. Biological soil crusts (BSCs) are complex ground cover formed mainly by algae, lichens, mosses, and a few soil microorganisms bound to soil surface particles through algae filaments, myceliua, rhizoids, and secretions [6]. BSCs are generally divided into four developmental stages: microbiotic, algae, lichen, and moss crusts. Among these, algal and moss crusts are the most common crust taxa in the successional process of desert vegetation, as well as the two communities with the highest biomass in BSCs, while also exhibiting physicochemical and biological properties that differ considerably from those of loose sandy soils. Hence, they can fulfill a variety of functions in sandy ecosystems, including acting as indicators of vegetation type, resistance to wind erosion, maintenance of soil stability, carbon sequestration, nitrogen fixation, and promotion of soil water circulation [7,8,9,10]. Therefore, selecting reasonable and effective sand control measures to promote the formation and development of BSCs can contribute to preventing the further spread of desertification. Studies have shown that different vegetation types in afforested areas can affect the growth and distribution of BSCs [11,12], whereas the formation and development of BSCs represent a key stage in the transformation of sandy land from mobile to fixed and semi-fixed dunes [13]. Hence, exploring the physicochemical properties and enzyme activities of different BSC types in areas undergoing different afforestation measures can, in turn, serve as important indicators for evaluating the recovery status of their soil ecosystems. Bacteria also play a critical role in the formation and development of BSCs and are involved in maintaining the structure and function of BSCs, as well as promoting ecosystem material cycling [14]. Therefore, studying the bacterial community composition of different BSC types under different afforestation measures can further elucidate the developmental mechanism of BSCs, which can facilitate the optimization of sand control strategies. He et al. [15] explored the differences in physicochemical properties and enzyme activities of different types of BSCs in the Tengger Desert. They reported that the nutrient contents and enzyme activities of BSCs exhibited a significant upward trend with the succession toward moss-dominated crust communities, providing evidence for a deeper understanding of the impact of BSC succession on the stability of desert ecosystems. In a study on Ordos sandy land, Cui et al. [16] reported that BSCs in *Sabina vulgaris* plantation fields exhibit much higher fine particle and nutrient contents than in other shrub fields, providing a strategy for the desertification control model of Ordos sandy lands. Similarly, in a study on Mu Us sandy lands, Zhang et al. [17] reported that the abundance and diversity of bacterial communities increase gradually with the successional stage from algal crust to lichen crust to moss crust. Their findings can help us further understand the successional mechanism during the development of BSCs, thereby providing a theoretical basis for BSC protection and ecological restoration in Mu Us sandy lands. Taken together, these studies demonstrate the importance of analyzing the physicochemical properties, enzyme activities, and bacterial community structures of different types of BSCs subjected to different afforestation measures for evaluating the ecosystem recovery of sandy land.

Alpine sandy land is a type of sandy land located at high altitudes (2500–3700 m), characterized by a cold and dry climate and low vegetation coverage [18]. Gonghe Basin is a typical alpine sandy land with an altitude of 2600~3400 m, low temperature, a poor natural environment, and serious desertification [19,20]. Currently, studies on BSCs in the Gonghe Basin have mainly focused on the effects of BSCs on soil physicochemical properties [21], the dynamics of soil carbon release under BSC cover [22], and the response characteristics of BSCs in climate simulations [23,24]. However, most of these studies are limited to the ecosystems of BSCs in specific plantation forest environments. Only Zhang et al. [25] have explored the effects of BSCs on soil physicochemical properties among three different shrub communities, but their study did not explore the enzyme activities and bacterial community structure of BSCs. This has hindered a comprehensive understanding of the effect of vegetation types on BSCs, which, in turn, has limited the precise optimization of ecological restoration strategies in alpine sandy lands. Therefore, to comprehensively explore the response characteristics of BSCs under different afforestation measures, this study more systematically assessed the effects of different afforestation measures on BSCs: (1) The differences in particle composition, physicochemical properties, and enzyme activities of bare sand (no crust cover, 0–2 cm soil layer), algal crust, and moss crust were investigated under four different afforestation measures. (2) High-throughput sequencing was employed to analyze the structure of bacterial communities in algal crust and moss crust under different afforestation measures. (3) Mantel test, redundancy analysis (RDA), and correlation heatmaps were used to illustrate the correlation among physicochemical properties, enzyme activities, and bacterial community structures in algal and moss crusts. In this study, we aimed to elucidate the response characteristics of two types of BSCs under different afforestation measures to further explore the mechanisms underlying the development of BSCs in alpine sandy lands. Our findings provide valuable insights that can be used as a reference for developing effective strategies to prevent and control sand drift in different BSC types across alpine sandy lands.

## 2. Materials and Methods

### 2.1. Study Area Profile

**Figure 1:**
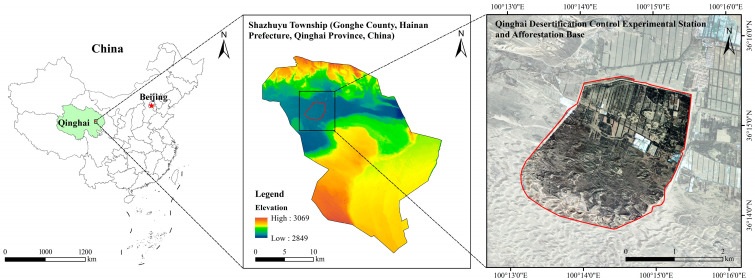
Study area profile.

This research was conducted in the Shazhuyu Sand Control Experimental Forest, Gonghe County, Qinghai Province (100°25′ E, 36°24′ N; Figure 1). Situated in the Gonghe Basin on the northeastern Tibetan Plateau, the site lies at an elevation of 2880 m. The region experiences a typical high-altitude continental climate, featuring cold, arid winters and springs. Climatic data indicate an annual mean temperature of 2.0–3.3 °C, with pronounced diurnal variations. Precipitation averages 264 mm/year, while annual evaporation reaches 1528–1937 mm. Persistent westerly/northwesterly winds drive intense wind erosion. The dominant soil type is aeolian sandy soil [19]. There are no naturally high forests in the study area. Shrubs in the area mainly include *Caragana korshinskii*, *Kalidium foliatum*, *Salsola arbuscula*, *Nitraria tangutorum*, and *Artemisia desertorum*, whereas herbaceous plants mainly include *Leymus secalinus* and *Stipa capillata*.

### 2.2. Biological Soil Crust Sample Collection

In late July 2024 (plant growing season), field surveys and biological soil crust sample collection were carried out in the study area. Four types of afforestation measures were selected: *Salix cheilophila* + *Populus simonii* plantation (WLYY), *Caragana korshinskii* plantation (NT80), *Salix psammophila* + *Salix cheilophila* plantation (SLWL), and *Artemisia ordosica* +*Caragana korshinskii* plantation (SHNT). Four afforestation projects were constructed in 1980, and the habitat conditions remained the same before construction. The four measures were implemented with relative success in the study area and were deployed on a large scale, showing good representativeness for similar types of sand control measures. When collecting the two types of crusts, reference [25] identified the algal crusts and the moss crusts. Algal and moss crusts were relatively common, whereas microbiotic and lichen crusts were rarely observed (Table 1). In each site, three 50 m × 50 m large sample plots (20 m × 20 m in the smaller WLYY site) and twelve 5 m × 5 m small sample plots were randomly established. Based on the principles of random and multi-point sampling, bare sand (no crust cover, 0–2 cm soil layer), algal crust, and moss crust samples were collected from each large sample plot, and samples of the same type were thoroughly mixed to yield one sample. Sampling was performed in triplicate for each of the three large sample plots to give a total of 36 samples. When collecting BSCs, a petri dish of 9.0 cm in diameter was used to mark the collection area, and a sterile shovel was used to separate the crust layer from the underlying soil. All sampling equipment was sterilized with 75% alcohol between different samples. In addition, the coverage and thickness of different BSC types and bare sand within each small sample plot were recorded and averaged across the 12 small sample plots to obtain representative values of coverage and thickness for different BSC types and bare sand.

### 2.3. Measurement of Biological Soil Crusts’ Physicochemical Properties and Enzyme Activity

The determination methods of each index are shown in Table 2.

### 2.4. 16S rDNA Extraction and Sequencing

Total bacterial DNA from soil samples was extracted using the DNA purification kit (MagaBio Soil GenomicDNA Purification Kit, Thermo Fisher Scientific, Shanghai, China). References [32,33] for pcr amplification and sequencing work.

### 2.5. Data Processing and Analyses

Based on the overlap relationship between paired-end (PE) reads, the FLASH 1.2.11 software was employed to splice and pair sequences. In addition, quality control was carried out using Fastp 0.19.6 software to obtain valid sequences. The significance of differences was tested using SPSS 27.0 (physicochemical properties, enzyme activity data, and bacterial community diversity index). OTU clustering was performed using Uparse 7.0.1090 software with a 97% similarity. The Mothur 1.30.2 software was used to calculate the Alpha diversity indices. The Kruskal–Wallis rank sum test and one-way ANOVA were performed to assess the differences in the relative abundance of dominant bacterial phyla in the same BSC type under different afforestation measures. The R language 3.3.1 was used to construct the plots for principal coordinate analysis (PCoA), bar charts for soil bacterial community composition, and correlation heatmaps. The boot and stats packages were used to test and plot the index differences between groups. The vegan package was used to construct RDA plots and Mantel test network heatmaps.

## 3. Results

### 3.1. Particle Composition of BSCs Under Different Afforestation Measures

**Figure 2:**
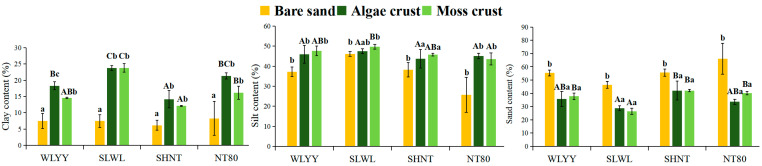
Particle composition of biological soil crusts (BSCs). Note: Data are represented as mean ± standard error; different capital letters indicate significant differences (*p* < 0.05) between the different sampling sites within the same BSC type; different lowercase letters indicate significant differences (*p* < 0.05) between the different BSC types within the same sampling site; WLYY: *Salix cheilophila* + *Populus simonii* plantation; SLWL: *Salix psammophila* + *S. cheilophila* plantation; SHNT: *Artemisia ordosica* + *Caragana korshinskii* plantation; and NT80: *C. korshinskii* plantation.

The particle composition of BSCs under different afforestation measures primarily consisted of silt and sand particles, which together accounted for 76–93% of the total particle composition (Figure 2). Among the four sites subjected to different afforestation measures, the algal and moss crusts of the SLWL plantations exhibited higher clay and silt contents than those in the same BSC types at the other plantation sites, whereas the opposite was true for sand particle contents between these plantation sites. Within the same plantation site, algal and moss crusts had lower sand content than those in bare sand, clay, and silt. Particle composition was more similar between the algal crust and the moss crust. These findings suggest that SLWL was better able to enrich fine particles, resulting in a higher content of fine particles in its BSC layer.

### 3.2. Physicochemical Properties and Enzyme Activities of BSCs Under Different Afforestation Measures

**Figure 3:**
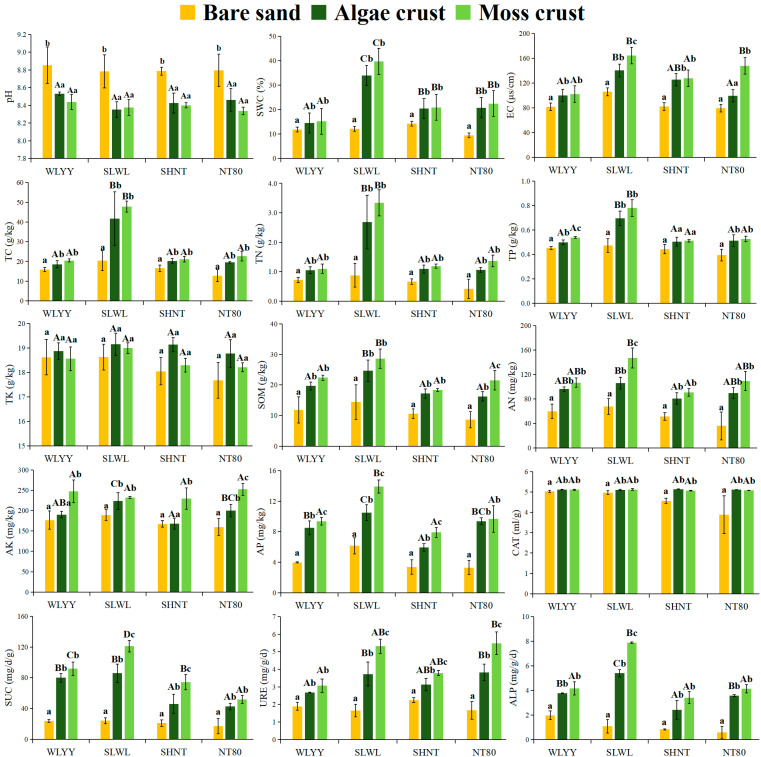
Physicochemical properties of biological soil crusts (BSCs) and their enzyme activities. Note: Data are represented as mean ± standard error; different capital letters indicate significant differences (*p* < 0.05) between different sampling sites within the same BSC type, and different lowercase letters indicate significant differences (*p* < 0.05) between the different BSC types within the same site; EC: electrical conductivity; SBD: soil bulk density; SWC: soil water content; TC: total carbon; TN: total nitrogen; SOM: soil organic matter; TP: total phosphorus; TK: total potassium; AN: alkaline-hydrolyzable nitrogen; AP: available phosphorus; AK: available potassium; CAT: catalase; SUC: sucrase; URE: urease; ALP: alkaline phosphatase; WLYY: *Salix cheilophila* + *Populus simonii* plantation; SLWL: *Salix psammophila* + *S. cheilophila* plantation; SHNT: *Artemisia ordosica* + *Caragana korshinskii* plantation; and NT80: *C. korshinskii* plantation.

Significant differences were found in physicochemical properties and enzyme activities within the same BSC type under different afforestation measures. Within the same site, the differences in physicochemical properties and enzyme activities between bare sand, algal crust, and moss crust were also relatively significant (Figure 3). Bare sand and BSCs under the four afforestation measures were alkaline. However, the pH values of algal and moss crusts were significantly lower than those of bare sand within the same site and no significant difference in pH values within the same type of BSC across different sample sites. Within the same sampling site, the SWC of both BSC types was significantly higher than that of bare sand, whereas the SWC of moss crust was also higher than that of algal crust, but the difference was not statistically significant. Among these, the SWC of BSCs at the SLWL sampling site was higher than that at the other sampling sites. Within the same sampling site, EC was ranked as follows: moss crust > algal crust > bare sand, whereas the EC of algal and moss crusts in SLWL was the highest within the same BSC type under the four afforestation measures. The characteristics of soil nutrient content showed that within the same site, the TC, TN, SOM, AN, and AP of algal crust and moss crust were significantly higher than those of bare sand, whereas the difference in AK between bare sand and BSCs was not statistically significant. The moss crust showed higher levels of TC, TN, TP, SOM, AN, AK, and AP compared to the algal crust, and only the TK content in the moss crust was lower than in the algal crust. Under the four afforestation measures, the SLWL plantation showed higher levels of TC, TN, TP, TK, SOM, AN, and AP in its algal and moss crusts than those at the other sampling sites, whereas the AK content in its algal crust was also higher than that in the algal crusts at the other sampling sites.

Within the same sampling site, CAT, SUC, URE, and ALP activities were significantly higher in both algal and moss crusts than in bare sand, whereas SUC, URE, and ALP activities were higher in moss crusts than in algal crusts. Under the four afforestation measures, SUC and ALP activities in algal and moss crusts at the SLWL sampling site were higher than those in the same BSC types at the other sampling sites. Moreover, URE activities in algal and moss crusts at the NT80 sampling site were higher than those in the same BSC types at the other sampling sites. There was virtually no difference in CAT activity within the same BSC type across the four different afforestation measures.

### 3.3. Correlation Between BSCs’ Physicochemical Properties and Enzyme Activity

**Figure 4:**
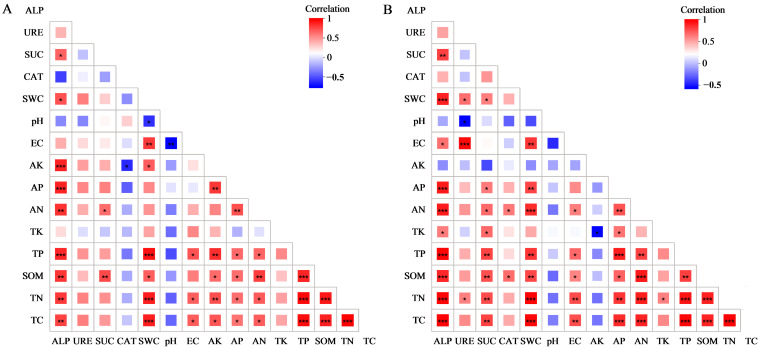
Heatmaps of correlations between biological soil crusts’ (BSCs) physicochemical properties and enzyme activities. Note: (**A**) is the heatmap of correlations between the physicochemical properties and enzyme activities in algal crust under different afforestation measures; (**B**) is the heatmap of correlations between the physicochemical properties and enzyme activities in moss crust under different afforestation measures; red in the heatmap indicates positive correlation; blue indicates negative correlation; the color depth represents the magnitude of positive or negative correlation, and asterisks in the colored squares indicate significance; * 0.01 < *p* ≤ 0.05, ** 0.001 < *p* ≤ 0.01, and *** *p* ≤ 0.001; EC: electrical conductivity; SWC: soil water content; TC: total carbon; TN: total nitrogen; SOM: soil organic matter; TP: total phosphorus; TK: total potassium; AN: alkaline-hydrolyzable nitrogen; AP: available phosphorus; AK: available potassium; CAT: catalase; SUC: sucrase; URE: urease; and ALP: alkaline phosphatase.

The Mantel test was performed to analyze the correlations between the physicochemical properties and enzyme activities of algal and moss crusts under different afforestation measures (Figure 4). As can be seen in Figure 4, the ALP of algal and moss crusts showed significant positive correlations with TC, TN, SOM, TP, AN, AP, and SWC; the ALP of moss crust showed significant positive correlations with TK and EC, and the ALP of algal crusts was significantly positively correlated with the content of AK. The URE activity of the algal crust did not correlate significantly with soil physicochemical properties or the activities of the other enzymes; however, the URE activity of the moss crust correlated significantly positively with TN, EC, and SWC and significantly negatively with pH. The SUC activity of the algal crust correlated significantly only with SOM and AN contents, whereas the SUC activity of the moss crust correlated significantly positively with AP, AN, TK, TP, SOM, TN, TC, and SWC. The CAT activity of the algal crust showed a significant negative correlation only with the AK content, whereas the CAT activity in the moss crust showed significant negative correlations only with SOM and AN contents. The results presented in Figure 4A,B demonstrate that among the four enzymes, only the activity of ALP correlated significantly with that of SUC. Among the soil physicochemical properties, SWC and EC exhibited significant positive correlations with most soil nutrient factors; pH had almost no significant correlation with other physicochemical properties, and most soil nutrient factors were mutually positively correlated, except TK and AK. Overall, the physicochemical properties and enzyme activities of moss crusts under the different afforestation measures were more closely correlated than those of algal crusts.

### 3.4. Bacterial Community Structure of BSCs Under Different Afforestation Measures

#### 3.4.1. Statistical Analysis of OTUs

**Figure 5:**
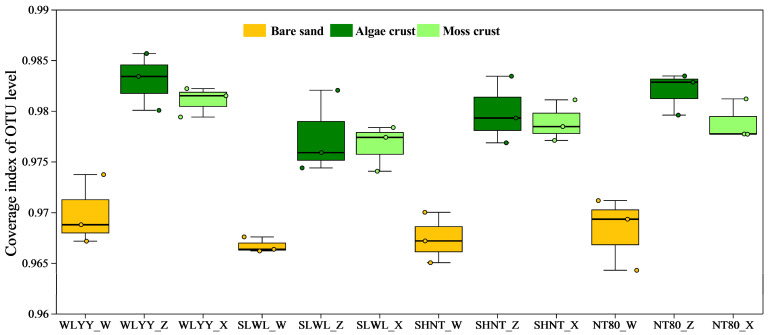
Bacterial community coverage. Note: WLYY: *Salix cheilophila* + *Populus simonii* plantation; SLWL: *Salix psammophila* + *S. cheilophila* plantation; SHNT: *Artemisia ordosica* + *Caragana korshinskii* plantation; NT80: *C. korshinskii* plantation; _W: bare sand; _Z: algal crust; _X: moss crust; and OTU: operational taxonomic unit.

A total of 1,786,860 quality-controlled sequences were obtained from all samples, with an average of 49,635 sequences per sample. After clustering and classification, 19,876 OTUs were identified, belonging to 1 kingdom, 43 phyla, 143 classes, 356 orders, 597 families, and 1196 genera. The coverage of all samples was above 0.95 (Figure 5), indicating that the sequencing results could accurately represent the true bacterial community structure in bare sand and the two BSC types under different afforestation measures.

#### 3.4.2. Soil Bacterial α-Diversity

**Figure 6:**
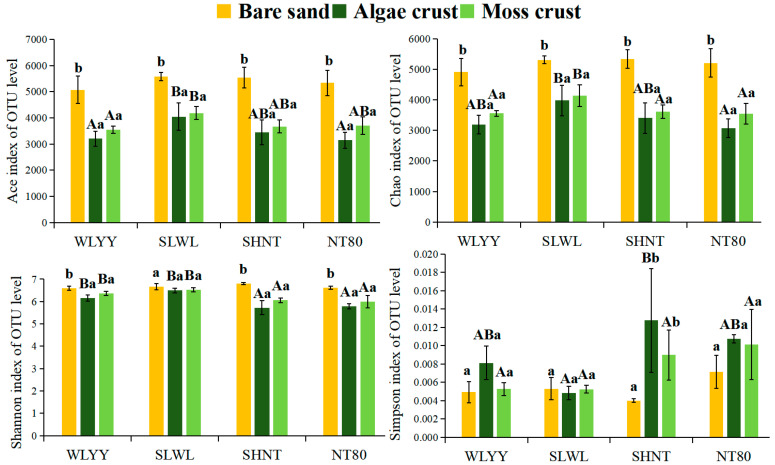
Bacterial α-diversity index. Note: Different uppercase letters indicate significant differences (*p* < 0.05) between the different sampling sites within the same BSC type. Different lowercase letters indicate significant differences (*p* < 0.05) between different BSC types within the same sampling site. WLYY: *Salix cheilophila* + *Populus simonii* plantation; SLWL: *Salix psammophila* + *S. cheilophila* plantation; SHNT: *Artemisia ordosica* + *Caragana korshinskii* plantation; NT80: *C. korshinskii* plantation; and OTU: operational taxonomic unit.

The α-diversity indices of bacterial communities in bare sand, algal crust, and moss crust were analyzed under different afforestation measures (Figure 6). Among these, the Chao and Ace indices are key indicators reflecting the richness of bacterial communities. The results revealed that within the same sampling site, the Ace and Chao indices of both BSC types were significantly lower than those of bare sand, whereas the Ace and Chao indices of moss crust were higher than those of algal crust, but the differences were not significant. These findings indicate that the richness of bacterial communities in BSCs was lower than that of bare sand without BSC cover, and the richness of bacterial communities in moss crust was higher than that in algal crust. Among the four afforestation measures, SLWL had the highest Ace and Chao indices for both algal and moss crusts, which suggests that the bacterial community richness of algal and moss crusts was more likely to increase under SLWL. The Shannon and Simpson indices are vital indicators of bacterial community diversity. At the SLWL sampling site, the Shannon and Simpson indices were relatively similar between bare sand and the BSCs. At the other three sampling sites, the Shannon indices of the bacterial communities were all ranked as follows: algal crust < moss crust < bare sand, whereas the Simpson indices of the bacterial communities were ranked in the following order: algal crust > moss crust > bare sand. This suggests that at the WLYY, SHNT, and NT80 sampling sites, the diversity of bacterial communities in algal and moss crusts was lower than that in bare sand, whereas the diversity of bacterial communities in moss crust was greater than that in algal crust; however, at the SLWL sampling site, the diversity of bacterial communities in bare sand and algal and moss crusts was relatively consistent. Among the four afforestation measures, SLWL revealed the highest Shannon and the smallest Simpson indices for the bacterial communities in algal and moss crusts. This entails that the diversity of bacterial communities in the algal crust and moss crust was more readily enhanced under SLWL.

#### 3.4.3. Composition of Soil Bacterial Communities

**Figure 7:**
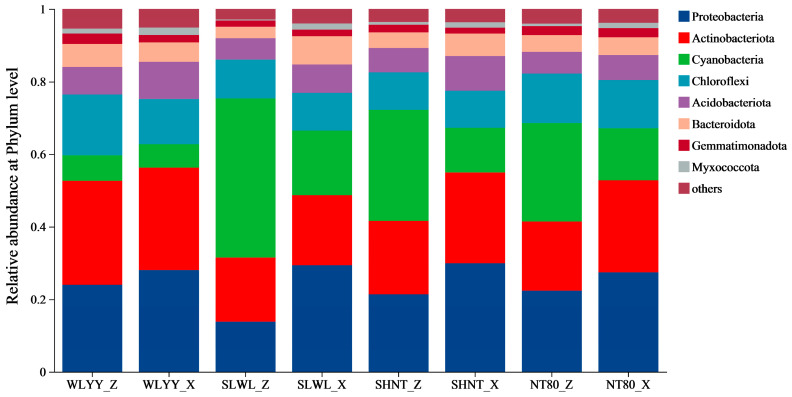
Composition of biological soil crusts (BSCs) bacterial communities. Note: WLYY: *Salix cheilophila* + *Populus simonii* plantation; SLWL: *Salix psammophila* + *S. cheilophila* plantation; SHNT: *Artemisia ordosica* + *Caragana korshinskii* plantation; NT80: *C. korshinskii* plantation; _Z: algal crust; and _X: moss crust.

At the phylum level, the soil bacterial communities of algal and moss crusts under different afforestation measures mainly include eight bacterial phyla (Figure 7). Among these, *Actinobacteriota*, *Proteobacteria*, *Acidobacteriota*, and *Chloroflexi* were the dominant phyla, accounting for 21.49–33.31%, 18.14–26.13%, 8.70–18.55%, and 10.91–15.63%, respectively. At each sampling site, the relative abundances of *Proteobacteria* and *Acidobacteriota* in the moss crust were higher than in the algal crust, whereas the relative abundance of *Cyanobacteria* in the moss crust was lower than that in the algal crust. This was particularly so at the SLWL and SHNT sampling sites, where the relative abundances of *Cyanobacteria* in the algal crust were relatively high, reaching 43.92 and 30.59%, respectively.

#### 3.4.4. PCoA of Soil Bacterial Communities

**Figure 8:**
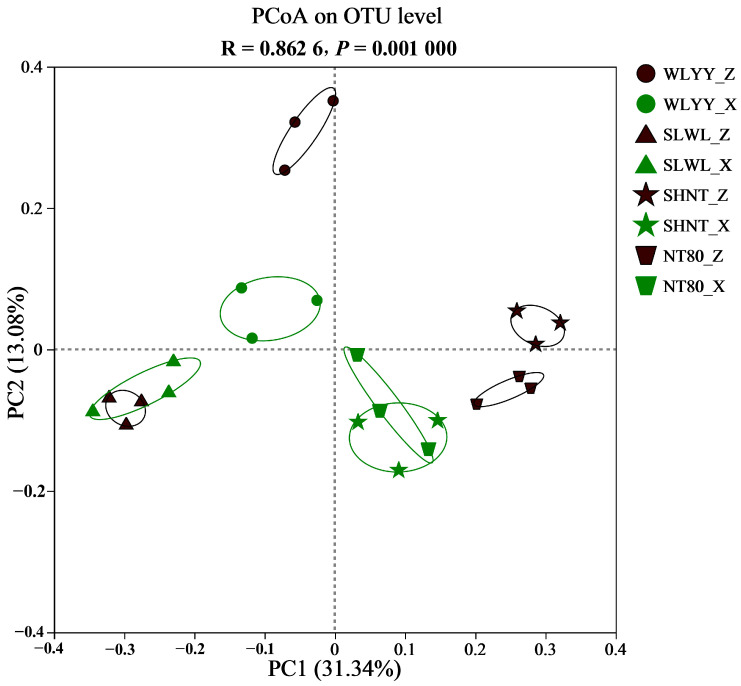
Principal coordinate analysis (PCoA) plot of bacterial community composition in biological soil crusts (BSCs) under the different afforestation measures. Note: WLYY: *Salix cheilophila* + *Populus simonii* plantation; SLWL: *Salix psammophila* + *S. cheilophila* plantation; SHNT: *Artemisia ordosica* + *Caragana korshinskii* plantation; NT80: *C. korshinskii* plantation; _Z: algal crust; _X: moss crust; OTU: operational taxonomic unit; and PC: principal coordinate.

PCoA was performed to analyze the similarities in the bacterial community composition of BSCs under different afforestation measures (Figure 8), and samples that are closer together indicate that they have greater similarity in community composition. PCoA (*R* = 0.8626, *p* = 0.0010) showed that both BSC types under SLWL and moss crust under WLYY were relatively clustered, suggesting that the bacterial community composition of algal and moss crusts in SLWL and moss crust in WLYY were relatively similar. The two types of BSCs were closer together in WLYY and SLWL and were farther apart in SHNT and NT80, indicating that the bacterial community composition of BSCs in WLYY was more similar to SLWL and more dissimilar to SHNT and NT80. The relative clustering of the two BSC types in NT80 and SHNT suggests a relatively similar bacterial community composition between the two sampling sites. This similarity could be attributed to the common vegetation *Salix cheilophila* shared between SLWL and WLYY *Caragan korshinskii* shared between NT80 and SHNT, causing SLWL and WLYY, as well as NT80 and SHNT, to have similar screening effects on the bacterial community.

#### 3.4.5. Between-Group Differences in Dominant Soil Bacterial Phyla

**Figure 9:**
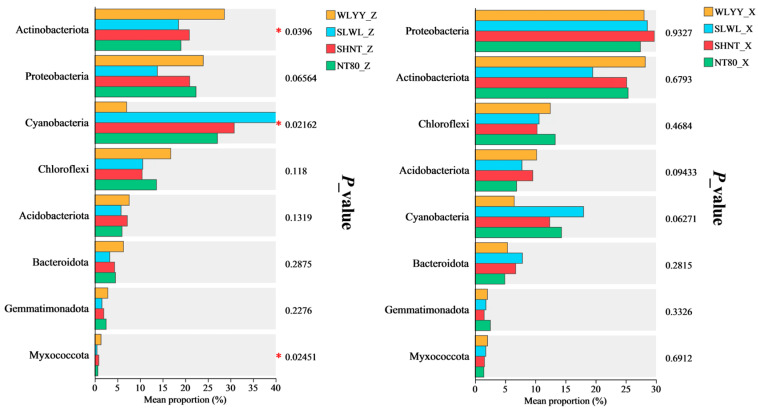
Tests of between-group differences in the bacterial communities of biological soil crusts (BSCs). Note: The eight bacterial phyla selected are the top eight dominant bacterial phyla in terms of relative abundance within the same type of BSC under different afforestation measures; asterisks denote significance; * 0.01 < *p* ≤ 0.05; WLYY: *Salix cheilophila* + *Populus simonii* plantation; SLWL: *Salix psammophila* + *S. cheilophila* plantation; SHNT: *Artemisia ordosica* + *Caragana korshinskii* plantation; NT80: *C. korshinskii* plantation; _Z: algal crust; and _X: moss crust.

Tests of between-group differences in dominant bacterial phyla were performed on the two types of BSCs under different afforestation measures (Figure 9). The results showed that in the moss crust, the relative abundances of the eight dominant bacterial phyla did not differ significantly between the sampling points. In the algal crust, the relative abundances of *Actinobacteriota*, *Cyanobacteria*, and *Myxococcota* differed significantly between sampling points, whereas the differences in the relative abundances of the remaining dominant bacterial phyla were not statistically significant. These results suggest that the relative abundances of dominant bacterial phyla in moss crust were relatively more stable under different afforestation measures, whereas the relative abundances of *Actinobacteriota*, *Cyanobacteria*, and *Myxococcota* in the algal crust were more sensitive to afforestation measures.

### 3.5. Correlations of BSCs’ Physicochemical Properties and Enzyme Activities with Bacterial Community Structures

#### 3.5.1. Correlations of Algal Crust Physicochemical Properties and Enzyme Activities with the Bacterial Community Structure

**Figure 10:**
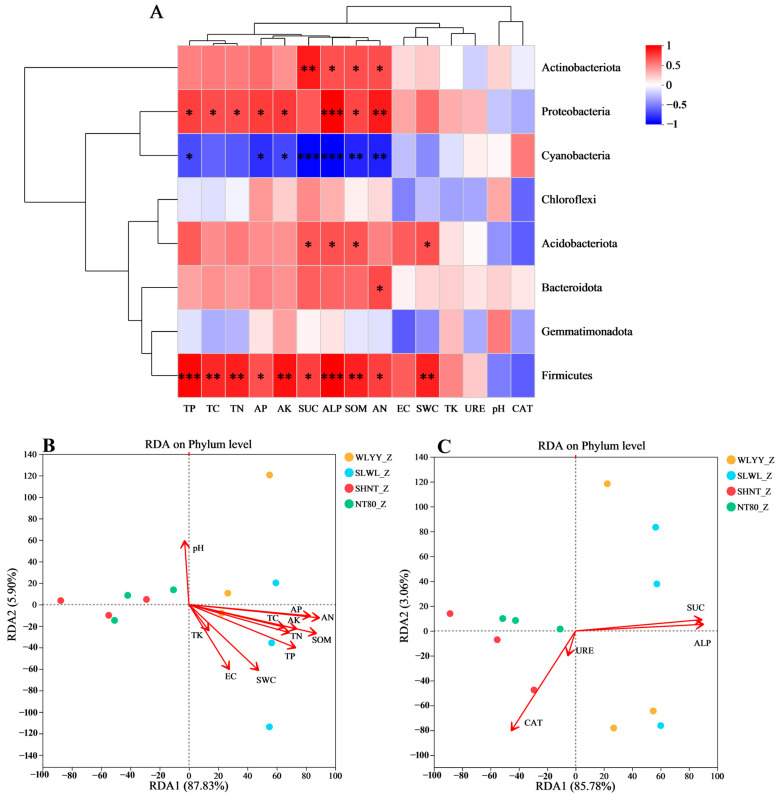
Correlations between algal crust physicochemical properties, enzyme activities, and bacterial community structures. Note: (**A**) is the heatmap showing the correlations of physicochemical properties and enzyme activities with the relative abundances of dominant bacterial phyla; the eight bacterial phyla selected are the top eight dominant bacterial phyla with respect to relative abundance in algal crust under different afforestation measures; (**B**) is the redundancy analysis (RDA) plot between physicochemical properties and bacterial community structure; (**C**) is the RDA plot between enzyme activity and bacterial community structure; WLYY: *Salix cheilophila* + *Populus simonii* plantation; SLWL: *Salix psammophila* + *S. cheilophila* plantation; SHNT: *Artemisia ordosica* + *Caragana korshinskii* plantation; NT80: *C. korshinskii* plantation; _Z: algal crust; red in the heatmap indicates positive correlation; blue indicates negative correlation; the color depth represents the magnitude of positive or negative correlations; asterisks in the colored squares indicate significance, * 0.01 < *p* ≤ 0.05, ** 0.001 < *p* ≤ 0.01, *** *p* ≤ 0.001; EC: electrical conductivity; SWC: soil water content; TC: total carbon; TN: total nitrogen; SOM: soil organic matter; TP: total phosphorus; TK: total potassium; AN: alkaline-hydrolyzable nitrogen; AP: available phosphorus; AK: available potassium; CAT: catalase; SUC: sucrase; URE: urease; and ALP: alkaline phosphatase.

Correlation analysis was performed on the physicochemical properties and enzyme activities of the algal crust with its bacterial community structure under different afforestation measures (Figure 10). We can see from Figure 10A that the relative abundance of *Actinobacteriota* had significant positive correlations only with SUC, ALP, SOM, and AN content, whereas the relative abundance of *Proteobacteria* exhibited significant positive correlations with TP, TC, TN, AP, AK, ALP, SOM, and AN content but not with other physicochemical properties and enzyme activities. Similarly, the relative abundance of *Cyanobacteria* correlated significantly negatively with TP, AP, AK, SUC, ALP, SOM, and AN contents but not with other physicochemical properties and enzyme activities, whereas the relative abundances of *Chloroflexi* and *Gemmatimonadota* showed no correlations with any of the physicochemical properties and enzyme activities measured in this study. Moreover, the relative abundance of *Acidobacteriota* showed significant positive correlations only with SUC, ALP, SOM, and SWC, whereas the relative abundance of *Bacteroidota* showed a significant positive correlation only with AN content but not with other physicochemical properties and enzyme activities. The relative abundance of *Firmicutes* showed significant positive correlations with TP, TC, TN, AP, AK, SUC, ALP, SOM, AN, and SWC but not with other physicochemical properties and enzyme activities. These results suggest that in the algal crust, the relative abundances of *Firmicutes*, *Proteobacteria*, and *Cyanobacteria* were more susceptible to the significant effects of physicochemical properties and enzyme activities than the other dominant bacterial phyla. Figure 10B shows the results of RDA for the relationship between soil physicochemical properties and bacterial community structures in the algal crust. The results demonstrated that among soil physicochemical properties, bacterial community composition structures were predominantly governed by AN in BSCs under different afforestation measures, followed by SOM, AP, and TP, in that order. Figure 10C shows the results of RDA for the relationship between enzyme activity and bacterial community structure in the algal crust. The results demonstrated that SUC and ALP exerted significant effects on the bacterial community structure in BSCs under different afforestation measures.

#### 3.5.2. Correlations Between Moss Crust Physicochemical Properties, Enzyme Activity, and Bacterial Community Structure

**Figure 11:**
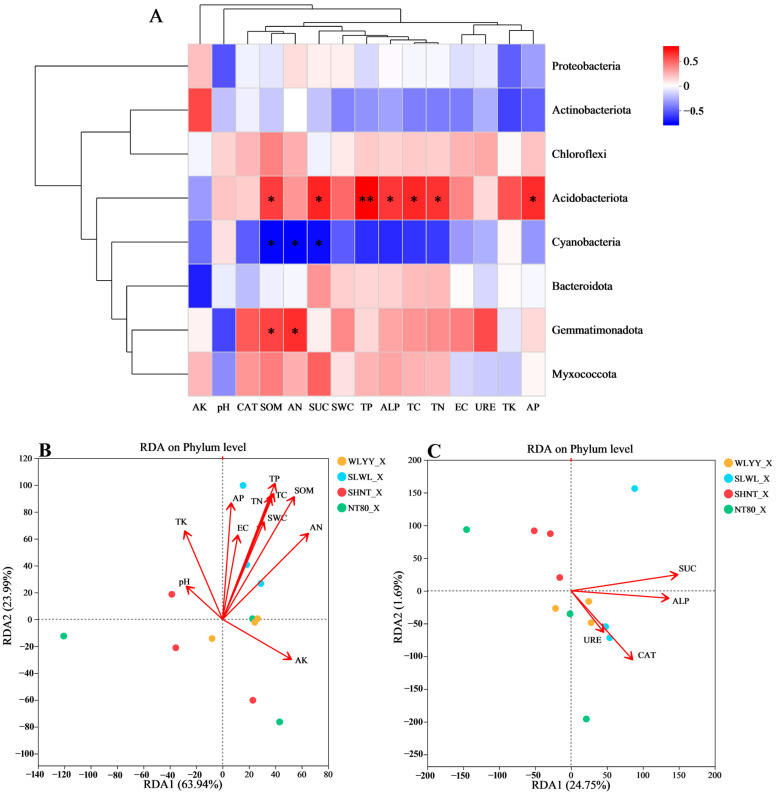
Correlations of moss crust physicochemical properties and enzyme activities with bacterial community structures. Note: (**A**) is the heatmap showing the correlations of physicochemical properties and enzyme activities with the relative abundances of dominant bacterial phyla; the eight bacterial phyla selected are the top eight dominant bacterial phyla with respect to relative abundance in moss crust under different afforestation measures; (**B**) is the redundancy analysis (RDA) plot between physicochemical properties and bacterial community structure; (**C**) is the RDA plot between enzyme activity and bacterial community structure; WLYY: *Salix cheilophila* + *Populus simonii* plantation; SLWL: *Salix psammophila* + *S. cheilophila* plantation; SHNT: *Artemisia ordosica* + *Caragana korshinskii* plantation; NT80: *C. korshinskii* plantation; _X: moss crust; red in the heatmap indicates positive correlation; blue indicates negative correlation; color depth indicates the magnitude of positive or negative correlation, and asterisks in the colored squares indicate significance; * 0.01 < *p* ≤ 0.05, ** 0.001 < *p* ≤ 0.01; EC: electrical conductivity; SWC: soil water content; TC: total carbon; TN: total nitrogen; SOM: soil organic matter; TP: total phosphorus; TK: total potassium; AN: alkaline-hydrolyzable nitrogen; AP: available phosphorus; AK: available potassium; CAT: catalase; SUC: sucrase; URE: urease; and ALP: alkaline phosphatase.

Correlation analysis was performed on the physicochemical properties and enzyme activities of moss crust with its bacterial community structure under different afforestation measures (Figure 11). Figure 11A shows that the relative abundances of *Proteobacteria*, *Actinobacteriota*, *Chloroflexi*, *Bacteroidota*, and *Myxococcota* showed no correlation with any of the physicochemical properties and enzymatic activities measured in this study; the relative abundance of *Acidobacteriota* correlated significantly positively with SOM, SUC, TP, ALP, TC, TN, and AP contents; the relative abundance of *Cyanobacteria* correlated significantly negatively with SOM, AN, and SUC but not with other physicochemical properties and enzyme activities; the relative abundance of *Gemmatimonadota* was significant positive correlations only with SOM and AN. These results suggest that the dominant bacterial phyla of moss crust under different afforestation measures were relatively more stable and less susceptible to significant effects of physicochemical properties and enzyme activities. Only the relative abundance of *Acidobacteriota* showed a high level of sensitivity to changes. Figure 11B shows that the first and second axes explained 63.94 and 23.99% of the variation, respectively. The results demonstrated that among soil physicochemical properties, AN was the main factor affecting the bacterial community structure in moss crust under different afforestation measures, followed by SOM, AK, and TP, in that order. Figure 11C shows the results of RDA for the relationship between enzyme activities and bacterial community structure in the moss crust. The results showed that SUC and ALP exhibited relatively large effects on the bacterial community structure in moss crust under different afforestation measures, which was consistent with the effects of enzyme activities on the bacterial community structure in algal crust, as described in Section 3.5.1.

## 4. Discussion

### 4.1. Effect of Different Afforestation Measures on BSCs Particle Composition

The alpine sandy lands of the Gonghe Basin are severely affected by wind erosion. In the absence of afforestation measures, the coverage of BSCs is greatly reduced, which will intensify soil erosion by wind and sand activities, thereby reducing fine particulate matter in topsoil [34]. The composition of soil particles can reflect the quality of soil texture, as well as characterize the degree of soil degradation and susceptibility to erosion [35,36]. In this study, the particle composition of both BSC types under four afforestation measures was dominated by silt particles. In a study also conducted on the Gonghe Basin, Zhang et al. [25] concluded that the particle composition of the BSCs was predominantly sand particles. This was because the measures selected for this study involved a longer period of restoration, which resulted in the greater development of BSCs and a larger accumulation of fine particles. In each sample site, the clay and silt contents of both BSC types were higher than those of bare sand, whereas the content of sand particles was lower than that of bare sand. This is consistent with the results by Guo et al. [37] on Horqin sandy lands. Elbert et al. [10] also found in their study of the Negev Desert that BSCs enhanced the retention of fine particles. This also showed that BSCs acted by refining the soil, and that the presence of BSCs led to the enrichment of finer particles and increased the resistance of soil to erosion, highlighting their universal role in soil stabilization across global drylands. Among the four afforestation measures, the content of fine particles was the highest in algal and moss crusts at the SLWL sampling sites, likely because both *S. psammophila* and *S. cheilophila* are bushy shrubs. More specifically, *S. psammophila* is shorter and more branched, whereas *S. cheilophila* has a larger crown and dense foliage. These morphologies can significantly reduce near-surface wind speed and effectively intercept clay, silt, and other fine particles that migrate with the wind. In addition, the litterfall of these two plants takes the form of fine twigs, which can readily be interwoven with surface sand particles to form a physical barrier, thus further fixing fine particles [25,38]. When compared among the four measures, *Populus simonii* has a relatively high crown; *Caragana korshinskii* has upright and sparse branches, and *Artemisia ordosica* is short but has limited coverage, all of which are inefficient at intercepting near-surface wind and sand flows, resulting in the reduced retention of fine particles. Therefore, among the four measures, SLWL can better facilitate soil refinement.

### 4.2. Effect of Different Afforestation Measures on BSCs’ Physicochemical Properties and Enzyme Activity

Soil salinization is a serious issue in land degradation that can lead to the deterioration of soil structure and loss of soil fertility. It is mainly manifested as excessively high soil salinity and alkalinity and usually occurs in arid and semi-arid areas [39,40,41]. In this study, the bare sand and both types of BSCs in the four afforested areas were alkaline, with no significant difference in pH between the two BSC types in each sample site, but both were significantly lower compared to bare sand. This may have been due to the secretion of acidic substances by BSCs through biological activities, as well as the accumulation of organic matter. Figure 4 also demonstrates that pH is negatively correlated with SOM, thereby improving the soil pH value, effectively slowing down the alkalization of soil, and promoting ecological restoration. The EC and SWC of both BSC types under the four afforestation measures were higher than those of bare sand. This was because the dense structure of the BSC layer slowed down the infiltration of water, resulting in a higher water content in the BSC layer, thereby further enhancing evaporation at the surface layer. This causes salt to rise with the water and be retained at the BSC layer, leading to an elevated EC [42]. It is important to note, however, that elevated EC values of BSCs can be detrimental to the colonization and reproduction of non-halophytic herbaceous plants, which may delay the recovery of desert ecosystems. The SWC of both BSC types in the SLWL area was significantly higher than that of the other sample sites. This was due to the higher content of fine particulate matter in the SLWL sample site, which is more likely to adsorb water in the soil by increasing its specific surface area. These findings also suggest that SLWL is more conducive to improving the water retention capacity of the BSC layer [43]. Soil nutrient content and enzyme activity in both BSC types were higher than those in bare sand under the four afforestation measures. Moreover, except for TK, the nutrient content and enzyme activity of moss crust were all higher than those of algal crusts. This is consistent with the results by He et al. [15] for the natural vegetation area of the Tengger Desert. This can mainly be attributed to the fact that crustal cryptogams in BSCs are closely associated with carbon and nitrogen fixation [44,45,46], and as bare sand transitions to the algal crust and then to moss crust, its photosynthesis and respiration will be further enhanced. All of this leads to differences in nutrients, such as organic matter, carbon, nitrogen, phosphorus, and so on [15,47], whereas BSCs can also capture atmospheric precipitation, thereby further promoting the accumulation of elements and increasing their nutrient content compared to bare sand [48,49]. The effect of BSCs on enzyme activity is also closely related to their cryptogamous characteristics. Moss crust can secrete more enzymes through rhizoids and litterfall, thus increasing their enzyme activities. With the succession of BSCs, there is an increase in darker pigments, which allow more solar radiation to be absorbed, thereby promoting an increase in soil temperatures. This is also beneficial for enhancing extracellular enzyme activities [44,50,51,52]. In general, the nutrient content and enzyme activity of the two BSC types were higher in the SLWL site compared to the other areas. This was also because the vegetation types could intercept fine particulate matter more effectively, promote the enrichment of fine particles in the BSC layer, and increase its water content to create a better environment for microbial activities, accelerate the decomposition of litterfall, hence increasing nutrient content and enzyme activity. Furthermore, in the SLWL site, *S. psammophila* and *S. cheilophila* may also directly enhance nutrient cycling through root secretions, which also suggests that SLWL is more favorable for promoting the increase in nutrient content and enzyme activity in the soil crust.

### 4.3. Effects of Different Afforestation Measures on the Bacterial Community Structure of BSCs

Microorganisms play an important role in the soil ecosystem [53], impacting soil structure by enhancing soil fertility. As soil microorganisms are sensitive to environmental changes, they can also serve as indicators for monitoring soil quality changes [54,55]. Among these microorganisms, bacteria account for the largest proportion, enabling them to occupy a dominant position in soil microbial communities and be widely employed as important indicators for evaluating soil quality [56,57]. In the two types of BSCs under the four plots in this study, the dominant bacterial phyla were Proteobacteria, Actinobacteriota, Cyanobacteria, and Chloroflexi, which were consistent with the results by Zhang et al. [17] in Mu Us sandy lands. Our findings also suggest that the dominant bacterial phyla of BSCs would not be altered, regardless of whether they were in an alpine environment. Across the different sample sites, the relative abundances of dominant bacterial phyla in the moss crust were more stable, whereas those of Actinobacteriota, Cyanobacteria, and Myxococcota in the algal crust were more sensitive to afforestation measures. This may be due to the high nutrient content of moss crust, which formed a state of “nutrient threshold saturation” in the bacterial community, resulting in their weak responses to nutrient differences caused by afforestation measures. In contrast, the algal crust had a lower nutrient content, and the relative abundances of specific bacterial phyla were susceptible to the nutrient differences regulated by afforestation measures, which manifests as significant adaptive divergence. In the WLYY, SHNT, and NT80 sample sites, bacterial community richness and diversity first decreased and then increased with the succession from bare sand to algal crust and then to moss crust. Moreover, the bacterial community richness and diversity of both BSC types were lower overall than those of bare sand. Zhang et al. [17] also hold this view in their research on the Mu Us Sandy Land. This was because, in bare sand, the competition between different bacteria was weak, and broadly adaptable bacteria could spread in large quantities, leading to higher bacterial community richness and diversity in bare sand. In the algal crust, the competitive inhibition of dominant bacterial species increased significantly, especially in the relative abundance of Cyanobacteria, which placed other species of bacteria at a disadvantage or even caused them to disappear during the competition. This led to a decrease in the richness and diversity of bacterial communities in the algal crust [58]. In the moss crust, the increase in nutrient content provided a better environment for bacterial growth. Hence, certain bacteria were able to reproduce and grow because of the rise in nutrient content, which led the moss crust to exhibit a higher richness and diversity of the bacterial community than in the algal crust. However, competitive inhibition by dominant bacterial species persisted, implying that the richness and diversity of the bacterial communities in algal and moss crusts were lower than in bare sand [59]. At the SLWL sampling site, despite the competition within the bacterial communities, the relatively high nutrient contents of the two BSC types eased the pressure of competitive inhibition and supported the synergistic coexistence of relatively more multifunctional bacterial communities. Hence, the richness and diversity of the bacterial communities were similar between bare sand and the two types of BSCs. This was the main reason why the bacterial community richness and diversity of the two BSC types were higher in the SLWL site compared to other sites. These results also show that SLWL is more favorable for increasing the richness and diversity of bacterial communities in BSCs.

In this study, AN and SOM were found to be the key physicochemical factors affecting the bacterial community structure of the algal crust and moss crust. Liu et al. [60] concluded in their study on the Kubuqi Desert that SOM and pH had the greatest influence on the bacterial community structure of the BSC layer, which suggests that the contribution of SOM to the bacterial community structure of BSCs is relatively stable. However, AN may be regulated by regional environmental characteristics, causing it to exert a greater impact on the bacterial community structure of BSCs. In addition, the environment of alpine sandy lands attenuated the modulatory effect of pH on the bacterial community structure of BSCs. This may have been due to the stronger ultraviolet radiation on alpine sandy lands, which altered the extracellular enzyme activity of the BSCs, thus further affecting the efficiency of bacterial nitrogen assimilation [52]. Among the enzyme activities of the two BSC types, SUC and ALP had a greater impact on bacterial community structure, most likely because SUC and ALP promote the soil carbon and phosphorus cycles, respectively [61,62], thus providing a source of readily available carbon and alleviating the pressure of phosphorus limitation for oligotrophic bacteria in alpine sandy lands, which alters the competitive advantage of bacteria with the corresponding metabolic pathways. Hence, SUC and ALP become the key enzymes driving the bacterial community structure of BSCs.

## 5. Conclusions

BSCs have a lower pH, finer particles, and higher water content than those in bare sand, which can effectively prevent further soil erosion and alkalization. During the succession from bare sand to algal crust and then to moss crust, nutrient content, and enzyme activities increased; bacterial community richness and diversity showed a general downward trend, followed by an upward trend, and the relative abundances of dominant bacterial phyla moved toward stability. Among the four afforestation measures, the SLWL measure can better promote the improvement in fine particle content, water content, nutrient content, enzyme activity, and the richness and diversity of bacterial communities in the crusts of algae and moss. AN and SOM were key factors influencing the bacterial community structure of BSCs under the four afforestation measures, while SUC and ALP also exerted a relatively large impact.

## 6. Patents

1. Shaobo Du, Huichun Xie, Chongyi E., Tianyue Zhao, Shuang Ji, Zhiqiang Dong, Shaoxiong Zhang, Haokun Wu. A plant fixation device for desertification control in deserts[P].utility model, 2024.7.26.
2. Shaobo Du, Huichun Xie, Chongyi E., Shujing Qi, Haokun Wu, Shuang Ji, Tianyue Zhao, Zhiqiang Dong. This invention relates to a portable spraying device for desert algae biological control of sand[P].utility model, 2025.3.28.

## Figures and Tables

**Table 1:** Basic information, including the biological soil crust (BSC) type of sample sites.

Site	Longitude	Latitude	Altitude (m)	Area (m^2^)	Vegetation Cover (%)	BSC Type	BSC Thickness (mm)	BSC Cover (%)
WLYY	100°15′19.0″ E	36°15′20.1″ N	2829	2500	82	Bare sand	-	11.24 ± 3.15
						Algal crust	9.14 ± 1.14	34.12 ± 6.17
						Moss crust	12.14 ± 1.12	54.63 ± 7.62
SLWL	100°14′16.9″ E	36°14′5.43″ N	2824	33,330	69	Bare sand	-	3.01 ± 1.04
						Algal crust	11.62 ± 2.15	36.58 ± 6.17
						Moss crust	13.16 ± 1.61	60.41 ± 8.14
SHNT	100°15′8.89″ E	36°14′41.2″ N	2817	19,998	66	Bare sand	-	19.27 ± 3.48
						Algal crust	5.7 7± 1.54	38.66 ± 6.62
						Moss crust	9.42 ± 0.46	42.08 ± 7.29
NT80	100°14′7.28″ E	36°14′55.7″ N	2811	10,000	89	Bare sand	-	13.68 ± 6.63
						Algal crust	8.15 ± 2.61	38.69 ± 4.49
						Moss crust	10.56 ± 0.74	47.61 ± 5.45

Note: Data are presented as mean ± standard error; “-” denotes no statistical significance; WLYY: *Salix cheilophila* + *Populus simonii* plantation; SLWL: *Salix psammophila* + *S. cheilophila* plantation; SHNT: *Artemisia ordosica* + *Caragana korshinskii* plantation; and NT80: *C. korshinskii* plantation.

**Table 2:** Indicator determination method.

Indicator	Method	Reference
Particle composition	laser diffraction analysis	[26]
pH	potentiometric method (water-to-soil ratio 2.5:1)	[27]
Electrical conductivity (EC)	conductometric method (water-to-soil ratio 5:1 for extraction)	[27]
Soil water content (SWC)	drying method	[27]
Alkaline-hydrolyzable nitrogen (AN)	alkaline hydrolysis diffusion	[27]
Available phosphorus (AP)	sodium bicarbonate extraction and molybdenum-antimony colorimetric	[27]
Total potassium (TK)	flame photometry method (Melting of NaOH)	[27]
Available potassium (AK)	flame photometry method (Extraction with 1 mol/L NH_4_OAc)	[27]
Total phosphorus (TP)	NaOH fusion and molybdenum-antimony colorimetric	[27]
Soil organic matter (SOM)	potassium dichromate-concentrated sulfuric acid external heating	[27]
Catalase (CAT)	potassium permanganate titration	[28]
Alkaline phosphatase (ALP)	disodium phenyl phosphate colorimetric	[29]
Urease (URE)	indole phenol acid colorimetric	[30]
Sucrase (SUC)	3,5-dinitrosalicylic acid colorimetric	[31]

## Data Availability

The original contributions presented in this study are included in this article; further inquiries can be directed to the corresponding authors.

## Abbreviations

The following abbreviations are used in this manuscript:

BSC	Biological soil crust
WLYY	*Salix cheilophila* + *Populus simonii* plantation
SLWL	*Salix psammophila* +*S. cheilophila* plantation
SHNT	*Artemisia ordosica* + *Caragana korshinskii* plantation
NT80	*Caragana korshinskii* plantation
